# Bismuth Sodium Titanate Based Materials for Piezoelectric Actuators

**DOI:** 10.3390/ma8125469

**Published:** 2015-12-04

**Authors:** Klaus Reichmann, Antonio Feteira, Ming Li

**Affiliations:** 1 Institute of Chemistry and Technology of Materials, Graz University of Technology, Stremayrgasse 9, Graz 8010, Austria; 2 Materials and Engineering Research Institute, Sheffield Hallam University, City Campus, Howard Street, Sheffield S1 1WB, UK; A.Feteira@shu.ac.uk; 3 Advanced Materials Research Group, Department of Mechanical, Materials and Manufacturing Engineering, The University of Nottingham, University Park, Nottingham NG7 2RD, UK; Ming.Li@nottingham.ac.uk

**Keywords:** piezoelectric actuator, multilayer, lead-free, bismuth sodium titanate

## Abstract

The ban of lead in many electronic products and the expectation that, sooner or later, this ban will include the currently exempt piezoelectric ceramics based on Lead-Zirconate-Titanate has motivated many research groups to look for lead-free substitutes. After a short overview on different classes of lead-free piezoelectric ceramics with large strain, this review will focus on Bismuth-Sodium-Titanate and its solid solutions. These compounds exhibit extraordinarily high strain, due to a field induced phase transition, which makes them attractive for actuator applications. The structural features of these materials and the origin of the field-induced strain will be revised. Technologies for texturing, which increases the useable strain, will be introduced. Finally, the features that are relevant for the application of these materials in a multilayer design will be summarized.

## 1. Introduction

Environmental regulations succeeded in replacing lead and lead components in a number of applications like paints, solder and a variety of electronic components. Exempt from this ban of lead are applications and technologies where no proper replacement is available, such as piezoelectric ceramics. These exemptions are limited in time and need to be discussed and renewed periodically. Concerning piezoelectric materials with its “workhorse” Lead-Zirconate-Titanate (PZT), this legal demand has triggered research efforts and materials development for some twenty years. A number of lead-free materials, supported by innovative processing technologies, like texturing, are competitive to replace this well establish compound. The discovery of an extraordinarily large strain generated by field-induced phase transition in Bismuth-Sodium-Titanate based solid solutions draw the attention of researchers to this material system, which seems to be an alternative to PZT for actuator applications. Actuators can be found in a variety of designs; the most common are bending actuators and stack actuators. Most of these ceramic devices are manufactured using multilayer technology, where ceramic layers are cofired with metal layers acting as inner electrodes. With this technology, it is possible to reduce the thickness of the ceramic layers to well below 100 μm, which enables generating a high electric field with a relatively low voltage applied. Combining this with a large number of layers, a high strain of the actuator can be achieved. A good example of a high-performance piezoelectric multilayer device is the stacked actuator for opening diesel injection valves in diesel engines, which consists of more than 300 single layers and has to cope with severe operating conditions (wide temperature range, high mechanical stress, large electric fields) as well as a high demand on reliability and lifetime. Therefore materials development needs to consider a lot more materials properties and issues than just the piezoelectric response.

Talking about lead-free ferroelectrics or piezoelectrics, respectively, with large strain, one may consider four distinct material classes, as presented in the review paper on lead-free piezoelectric ceramics by Rödel, *et al.* [1]. The first is based on BaTiO_3_ (BT). Solid solutions with CaTiO_3_ and BaZrO_3_, commonly denoted as BCZT [2,3,4,5,6], achieve extraordinarily high strain (exceeding that of PZT) in a temperature range around room temperature. The limiting Curie Temperature (*T*_C_) is approximately 100 °C. The second class, based on K_0.5_Na_0.5_NbO_3_ (KNN), exhibits lower strain but the Curie Temperature may reach 450 °C, which is beyond that of PZT-based compounds [1,7,8,9,10]. The third group are ferroelectrics based on Bi_0.5_Na_0.5_TiO_3_ (BNT) [11,12,13,14], where the limiting temperature is not a more or less diffuse Curie Temperature, but a so-called Depolarization Temperature (*T*_d_), whose maximum is around 290 °C and most doping agents lower that *T*_d_. The fourth class comprises so-called BNT-based incipient piezoelectrics [15,16,17,18,19,20,21]. In such materials, e.g., the solid solution of BNT and SrTiO_3_ [15], one can gain giant strains, which are caused by a reversible phase transition from an ergodic relaxor state to a ferroelectric state induced by the electric field applied. This is the class of materials that will be found in most examples given later.

Looking back, it might be justified to say that the greatest push and encouragement for materials scientists in that field came from the publication of Saito, *et al.* [22] in 2004 and by the two papers from Zhang, *et al.* [18,19] in 2008. Saitos publications showed that the inferior piezoelectric properties of KNN compared to PZT can be raised to the level of PZT by applying a technology that provides a highly textured ceramics. Therefore, the effect of texturing will be considered in its own section in this paper. Zhang’s findings of a field induced giant strain in BNT-BT-KNN and its temperature dependence encouraged other researchers to regard the Depolarization Temperature in that system not as a limit but just as a border to a new “land of hope”, where new materials for lead-free piezoelectric actuators can be found.

This contribution will give a short overview of lead-free piezoelectric ceramics with large strain and will introduce the Bismuth-Sodium-Titanate compound and some of its derivatives summarizing their structural peculiarities and the current opinion on the origin of the large field-induced strain. Furthermore, this review will add aspects of the processing technology of texturing, which is able to increase usable strain of these materials and, even more important for energy conversion, coupling factor. In the following sections, the features of a piezoelectric actuator material are outlined, such as blocking force, temperature dependence of strain, and interaction with electrode materials during co-firing, that have to be considered for a useful and reliable device. Examples of material systems used for multilayer actuators, which are described in literature, are provided.

## 2. Structure of BNT and Its Solid Solutions

### 2.1. Room Temperature Structure

The interesting ferroelectric and piezoelectric properties of BNT have triggered extensive studies on its complex perovskite-based crystal structure using a wide range of experimental techniques and simulations. However, to date both the space group at room temperature (RT) and the nature of high temperature phase transitions are still under debate.

The early studies in the 1960s using X-ray diffraction (XRD) suggest BNT adopts either pseudo-cubic [23,24] or rhombohedral [25] symmetry. It was proposed in a later XRD study [26] that the space group is R3m, which is polar but without octahedral tilting (*a°a°a°*, according to the Glazer notation [27]). A subsequent neutron diffraction study revealed the presence of superlattice reflections associated with doubling of unit cell, indicating the space group is R3c [28]. The R3c model was adopted in another more detailed neutron diffraction study [29]. In the R3c phase, the cations are displaced parallel to [111] *p* with the *a*^−^*a*^−^*a*^−^ antiphase rotations of the TiO_6_ octahedra about the threefold pseudo-cubic axes.

However, there is also experimental evidence that is inconsistent with the R3c symmetry. The temperature dependence of birefringence and opalescence data suggest a lower than rhombohedral symmetry [30]. An X-ray diffuse scattering study suggests Bi could move off the [111] *p* axis to improve its coordination environment and consequently reduce the local symmetry to be monoclinic [31]. The X-ray absorption fine structure (XAFS) results [32] show very different Bi–O distances from those refined using the neutron diffraction data. XAFS results reveal the shortest Bi–O distance to be ~2.2 Å, consistent with that observed in most bismuth oxides. However, the refined structure using the neutron diffraction data with the R3c symmetry leads to severely under-bonded Bi coordination environment with shortest Bi–O distance being more than 2.5 Å. This study suggests the local environment of Bi is much more distorted than that expected from the average structure. A neutron scattering study shows the rhombohedral phase has incommensurate modulation along the four-fold axis of a tetragonal phase [33], which is presumably caused by local Na and Bi ordering. More recent X-ray and neutron total scattering experiments and pair distribution function (PDF) analysis [34,35] demonstrate the complex local structure in BNT. There are distinct Na and Bi cation displacements as well as two different Bi positions [34].

**Figure 1:**
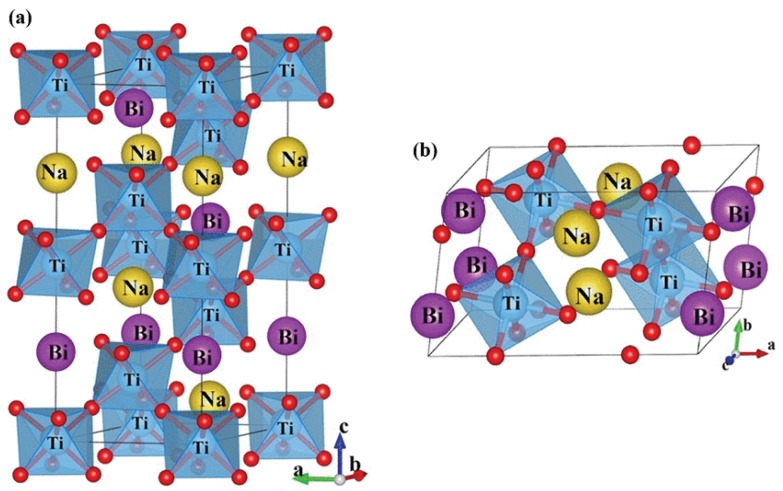
Crystal structure models of (**a**) rhombohedral R3c (hexagonal setting); and (**b**) monoclinic Cc phases (from reference [39], Copyright from The American Physical Society 2013).

Based on the high-resolution single-crystal XRD data, a monoclinic Cc model was proposed [36]. This model was then adopted in the subsequent high-resolution powder XRD studies [37,38]. The Cc phase has very similar lattice parameters [36] (*a* = *b* = 3.887, *c* = 3.882, α = β = 89.944, γ = 89.646) as compared to the R3c phase (*a* = *b* = *c* = 3.885, α = β = γ = 89.83) but has a different octahedra tilting system of *a*^−^*a*^−^*c*^−^. The tilting is still out-of-phase in the *x*, *y*, and *z* directions but the tilting angel is different in the *z*-direction. Figure 1 show the crystal structure of the two models. The R3c model could fit the synchrotron X-ray patterns collected using calcined powders but peak splitting is observed in the sintered samples [37]. The Cc model gives better fit than the R3c model. Nevertheless, it is still not completely satisfactory. It does not resolve the issue of under-bounded Bi environment [36] and could not model small variations in the diffraction patterns associated with local disorder [38]. The monoclinic (Cc) + cubic (Pm$\bar{3}$m) model was employed to fit the XRD patterns for the sintered samples in the previous report [38].

Based on a combined study using electron, X-ray, and neutron diffraction coupled with first principles calculation, and the dielectric, ferroelectric, and piezoelectric measurements [39,40,41], it is proposed that the Cc and R3c phases coexist at RT in undoped samples. The relative fraction of the two phases depends on thermal, mechanical, and electrical stimuli. Under external electric field the Cc phase is irreversibly suppressed and the XRD patterns of poled BNT samples could be nicely fitted with the R3c model alone [39,40,41]. It is therefore concluded that the Cc phase is not a new equilibrium phase. Instead, it is associated with local structural and strain heterogeneities. External electrical filed can remove such heterogeneities and the symmetry becomes purely rhombohedral. A transmission electron microscopy (TEM) work [42] shows defect-free BNT has an average R3c symmetry on length scales of a few nanometers. The bulk BNT contains various types of defects including antiphase boundaries, domain walls, and tetragonal platelets. The Cc phase is present only in the vicinity of domain walls.

TEM work also reveal the presence of nanometer scale tetragonal platelets with *a°a°c^+^* tilting system [43,44]. It is proposed BNT consists of two phases with tetragonal *a°a°c^+^* platelets inhomogeneously distributed in the *a*^−^*a*^−^*a*^−^ rhombohedral matrix. A different explanation of the electron diffraction data is that the structure can be best described by a “continuous tilt” model [45]. Nanoscale domains with *a^−^a^−^c^+^* tilting are limited to a length of a few unit cells and assemblages of such nanodomains exhibit an average *a^−^a^−^c^−^* tilting system. This continuous tilting model is consistent with the “average” monoclinic structure. The Cc phase is observed in another TEM work [46]. The authors also stressed that, as TEM is a local structure technique, such results do not rule out the existence of other phases.

Apart from the complex cation displacements and oxygen octahedra tilting, possible Na and Bi ordering is another factor adding further complexity to the crystal structure. In perovskite oxides, larger difference in charge and size generally leads to cation ordering whereas similar charge and size of cations give disordered structure [47,48]. The ionic radii of Na are close to that of Bi [49], implying long range ordering is unlikely. The charge difference between Na^+^ and Bi^3+^ is not large enough to guarantee long range A-site cation ordering. Simulation results [50] suggest the calculated ordering energies are small as compared to the thermal energy and only local ordering is possible. Experimentally there are inconsistent evidences on the A-site cation ordering. X-ray and neutron data reveal no superstructure peaks associated with long range Na and Bi ordering [29,35]. In one report [51], however, it is claimed, based on the single crystal XRD data, long range ordering exists but is very weak. Raman spectroscopy data [52,53] support the existence of local Na/Bi ordering. However, TEM data [45] show no evidence of local Na/Bi ordering.

### 2.2. High Temperature Phase Transitions

BNT exhibits so-called “peculiar” ferroelectric behaviors. The temperature dependence of relative permittivity (ε*_r_*) shows a broad maximum at ~320 °C (*T*_m_) and a small hump at ~200 °C [23,54]. Linear thermal expansion coefficient also shows noticeable changes at the temperature range of 200–320 °C [23]. A large hysteresis of 50–60 °C for various physical properties including dielectric [55], thermal [55] and optical [56] properties is observed at 200–320 °C. Extensive efforts have been made to probe the structural origins associated with the temperature dependence of the physical properties [26,29,38,52,55,57,58,59,60,61,62,63,64,65,66]. The long outstanding puzzle remains that the average structure obtained using X-ray and neutron diffraction [29,38] does not coincide with the physical properties.

It is generally believed that BNT exhibits at least two phase transitions between RT and 520 °C [26,29,38,52,55,57,58,59,60,61,62,63,64,65,66]. On cooling, BNT transforms from cubic to tetragonal polymorphism (space group P4bm) at ~520 °C and to rhombohedral polymorphism at ~250 °C [29]. Rhombohedral and tetragonal polymorphisms coexist at ~250–400 °C [29,63]. BNT adopts a cubic structure (space group Pm$\bar{3}$m) above ~520 °C. The phase transition at 520 °C is of first order. Differential scanning calorimetry (DSC) reveals a maximum of specific heat on heating and a minimum on cooling [55]. A maximum of linear thermal expansion coefficient is also observed [55]. However, no obvious permittivity anomaly observed at ~520 °C. A recent study [67] reveals a loss peak at ~500 °C in highly-insulating Nb-doped BNT samples. Undoped BNT samples typically exhibit high leakage current at this temperature range. Therefore the loss peak could be masked by the high loss background and becomes invisible. The loss peak at ~500 °C could be associated with the cubic-tetragonal phase transition but further study is needed to confirm this.

The most intriguing behavior occurs at 200–320 °C. X-ray and neutron diffraction [29,38] reveal no phase transition at 320 °C where ε*_r_* exhibits a maximum and at 200 °C where BNT loses polarization. One early study [57] suggests BNT is antiferroelectric at 200–320 °C. On cooling it exhibits paraelectric-antiferroelectric phase transition at 320 °C and antiferroelectric-ferroelectric phase transition at 200 °C. Double hysteresis loops, a typical characteristic of antiferroelectrics, are presented as the evidence [57]. However, no superlattice reflection associated with antiparallel cation displacements is observed by X-ray and neutron diffraction and Raman data [29,38,60].

**Figure 2:**
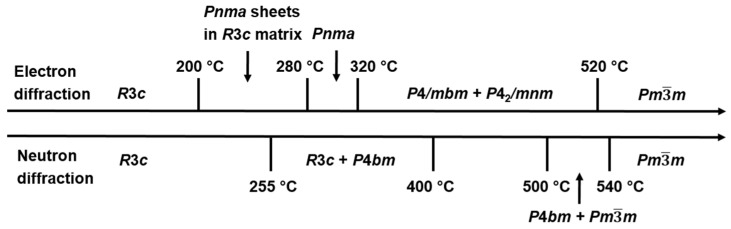
Reported possible phase transition routes based on TEM and X-ray/neutron diffraction studies (data from references [29,68,69]).

High temperature *in-situ* TEM studies [68,69,70] provide an explanation for the ferroelectric behaviors at 200–520 °C. The rhombohedral to tetragonal phase transition upon heating involves two processes and the formation of an intermediate orthorhombic Pnma phase. An intermediate modulated phase consisting of R3c blocks periodically separated by orthorhombic Pnma sheets starts to grow slightly above 200 °C through a micro twining process. With increasing temperature the volume fraction of orthorhombic Pnma sheets increases. At ~300 °C the orthorhombic Pnma phase forms that then transforms into the tetragonal polymorphism (P4/mbm) at 320 °C. Note that the orthorhombic phase has not been observed by X-ray/neutron diffraction studies. The possible routes of high temperature phase transitions based on TEM and X-ray/neutron diffraction are summarized in Figure 2 [29,68,69].

In summary, BNT exhibit extremely complex crystal structure caused by distinctly different displacement of Na and Bi ions, complex octahedral tilting, possible local A-site cation ordering as well as local deviations from global structure due to various defects that are sensitive to external stimuli. In addition, the deviations from cubic structure are small [36]. For example, the RT rhombohedral angle is 89.83°, very close to 90°. For the tetragonal phase, *c/a* is 1.004. All these factors, especially when combined together, make it tremendously challenging to reveal the true symmetry of BNT.

### 2.3. Phase Diagrams of Solid Solutions

Piezoelectric materials exhibit enhanced properties with compositions around the so-called morphotropic phase boundary (MPB) regions [71]. For the well-known binary phase PbZr_1-*x*_Ti*_x_*O_3_ (PZT) system, the MPB lies at *x* = 0.48. For *x* < 0.48, PZT adopts the rhombohedral R3c symmetry. For *x* > 0.48, PZT adopts tetragonal P4mm symmetry. For BNT, various solid solutions with other ferroelectric materials including BaTiO_3_ (BT), Bi_1/2_K_1/2_TiO_3_ (BKT) and K_1/2_Na_1/2_NbO_3_ (KNN) have been developed to obtain MPBs for better piezoelectric properties [16,72,73,74,75,76,77,78,79,80,81,82,83,84,85,86,87,88,89]. The approach is to combine the rhombohedral BNT with some tetragonal compounds (e.g., BT, BKT) to create MPB compositions (similar to the case of PZT). Addition of other components just increases the diffuseness of phase transitions.

**Figure 3:**
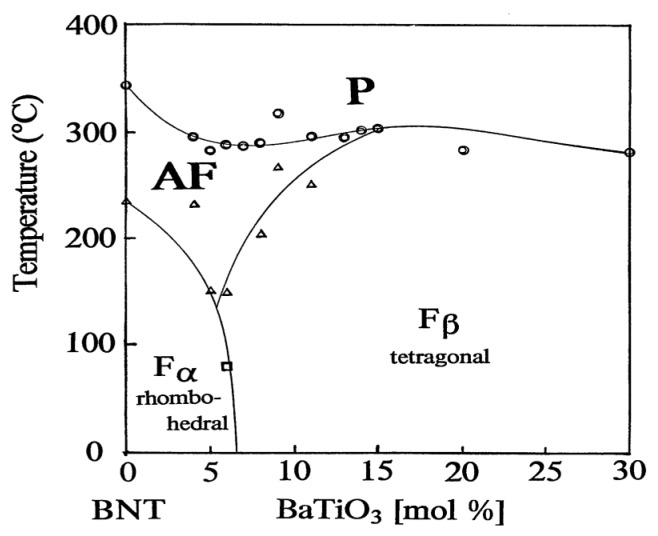
Morphotropic phase boundary in BNT-based solid solutions with BaTiO_3_, for poled ceramics ([72], Copyright from The Japan Society of Applied Physics 1991).

The phase diagram of the binary system (1−*x*) Bi_1/2_Na_1/2_TiO_3_ – x BaTiO_3_ (BNT-BT), Figure 3a, reveals the presence of an MPB between ferroelectric rhombohedral and tetragonal phases at *x* close to 0.06–0.08 [72]. The existence of MPB at this composition appears evident based on enhanced piezoelectric properties. The exact structure at MPB is however still under debate [80,81,82,83]. The predicted MBP using *ab* *initio* calculations lies at *x* = 0.35 [90], very different from the experimental values, which is presumably caused by temperature effects associated with 0 kelvin approximation of the calculation and uncertainty in the experimental data [90]. Another MPB at *x* = 0.03–0.04 was recently found [46], which separates the monoclinic Cc and rhombohedral R3c phases. It was also reported that electrical poling can create, destroy, or even replace the MPB with another one in BNT-BT [91].

There have been inconsistent reports on the MPB in the (1−*x*) Bi_1/2_Na_1/2_TiO_3_ – *x* Bi_1/2_K_1/2_TiO_3_ system (BNT-BKT). MPB lies at *x* close to 0.16–0.20 [73]. Broader composition range (*x* = 0.08–0.3) was also reported [92]. There are also reports claiming no coexistence of rhombohedral/tetragonal phases in BNT-BKT single crystals [93,94]. It is noted the final compositions, as analyzed using X-ray fluorescence (XRF) and inductively coupled plasma—optical emission spectroscopy (ICP-OES), show clearly a much lower potassium content than the nominal values. The final compositions are different in the two reports [93,94], suggesting sample processing conditions may significantly influence the final compositions. This may explain the reported discrepancies in the literature.

The ternary system BNT–BT–KNN (ratio: 92/6/2) exhibits giant strain [77]. The optimised BNT-BT-BKT (ratio: 85.2/2.8/12) system combines a relative high Curie temperature and high piezoelectric constant [88].

### 2.4. Nonstoichiometry and Defect Chemistry

**Figure 4:**
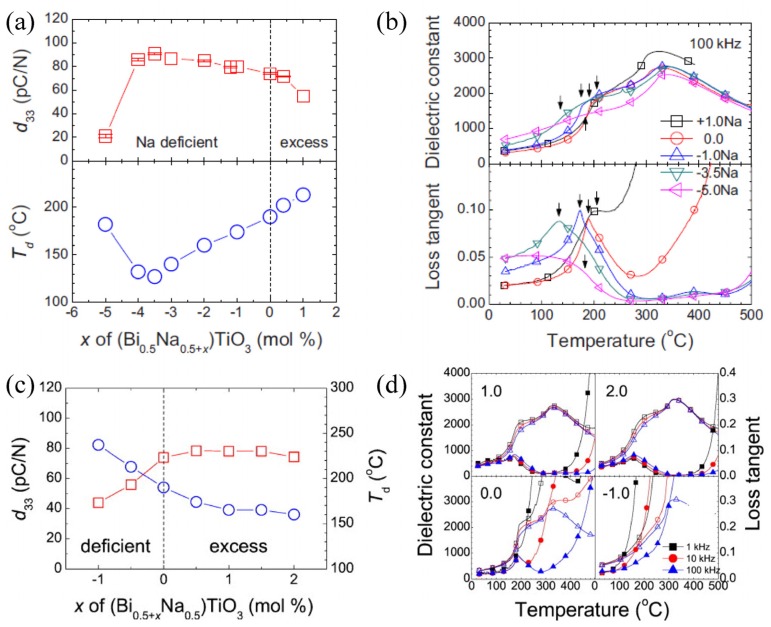
Influence of (**a**,**b**) Na and (**c**,**d**) Bi nonstoichiometry on depolarization temperature (*T*_d_), d_33_, relative permittivity and loss tangent (Reprinted with permission from references [96,97] Copyright from American Institute of Physics 2010, 2011).

Small compositional deviations of either A-site cations or Ti cation have been shown to influence the crystal structure, ceramic microstructure and piezoelectric properties [51,54,95,96,97,98,99,100,101]. Either Na deficiency or Bi excess in the nominal starting composition enhances the dc resistivity, d_33_ but lowers *T*_d_, octahedral tilt angle and ceramic grain size [54,96,97,98]. On the other hand, either Na excess or Bi deficiency lowers the RT dc resistivity, d_33_ but enhances *T*_d_, octahedral tilt angle and ceramic grain size [54,96,97,98]. Figure 4 shows the variations of d_33_, *T*_d_, relative permittivity and loss tangent with Na and Bi stoichiometry.

It is also noted that the dielectric loss of compositions with Na deficiency or Bi excess is very low (<0.01) at 300–500 °C, whereas the dielectric loss of compositions with Na excess or Bi deficiency increases sharply from ~200 °C. The changes of RT dc resistivity and the dielectric loss imply the A-site cation nonstoichiometry changes bulk electrical conductivity.

The electrical conduction mechanisms in BNT were recently clarified using a combination of impedance spectroscopy, electromotive force and ^18^O tracer diffusion measurements [102,103]. BNT shows very different electrical behavior as compared to other titanates such as (BaSr)TiO_3_ in that BNT exhibit high oxide ion conductivity (depending on Na/Bi stoichiometry) whereas conductivity in (BaSr)TiO_3_ is dominated by electronic conduction. It is well known that low levels of nonstoichiometry (<1 at %) could lead to significant changes of electronic conductivity in (BaSr)TiO_3_ [104]. The nonstoichiometry may be associated with deliberate chemical doping, unintentional element loss/gain during sample processing and impurities. Small compositional variations also induce significant changes in the electrical conductivity in BNT, which however has a different origin and is related to a switch between electronic conduction and oxygen ion conduction [102,103].

**Figure 5:**
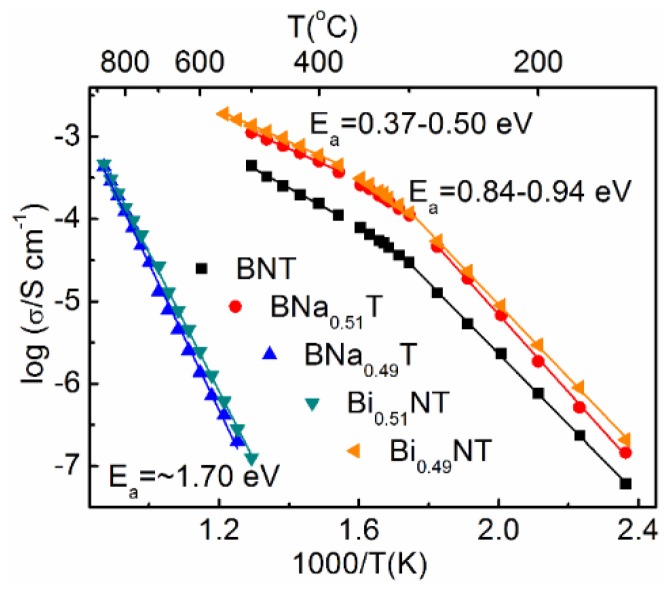
Arrhenius-type plots of bulk conductivity for nominal compositions Bi_0.5_Na_0.5_TiO_3_ (BNT), Bi_0.49_Na_0.5_TiO_2.985_ (Bi_0.49_NT), Bi_0.51_Na_0.5_TiO_3.015_ (Bi_0.51_NT), Bi_0.5_Na_0.49_TiO_2.995_ (BNa_0.49_T) and Bi_0.5_Na_0.51_TiO_3.005_ (BNa_0.51_T) (from references [102,103]).

The Arrhenius-type plots of bulk conductivity (Figure 5) show the BNT-based samples with slightly different starting compositions can be divided into two groups [102,103]. Nominal compositions with Bi excess (Bi_0.51_NT) or Na deficiency (BNa_0.49_T) exhibit low conductivity with an activation energy (*E*_a_) for bulk conduction of ~1.7 eV. The reported optical band gap (*E*_g_) for BNT is in the range of 3.0–3.5 eV [105,106,107], suggesting the electrical conduction is close to/dominated by intrinsic electronic conduction where *E*_g_ ~ 2*E*_a_. The nominally stoichiometric BNT and the compositions with Bi deficiency or Na excess exhibit much higher conductivity (by more than three orders of magnitude at temperatures below 600 °C). The *E*_a_ decreases to 0.8–0.9 eV and ~0.4–0.5 eV for the temperature ranges below and above the temperature associated with the maximum in ε*_r_* (*T*_m_ ~ 320 °C), suggesting a change in electrical conduction mechanism. A combination of impedance spectroscopy, electromotive force (EMF) and ^18^O tracer diffusion measurements confirm the higher conductivity is associated with oxygen ion conduction [102,103]. The high oxygen ion conductivity is attributed to high oxygen ion mobility associated with highly polarized Bi^3+^ cations and weak Bi–O bond [108] as well as oxygen vacancies generated together with bismuth vacancies:

(1) $$2{\text{Bi}}_{\text{Bi}}^{\times }\;+\;3{\text{O}}_{\text{O}}^{\times }\;\to \;2{\text{V}}_{\text{Bi}}^{\prime \prime \prime }+3{\text{V}}_{\text{O}}^{••}\;+\;{\text{Bi}}_{2}{\text{O}}_{3}$$

For the nominal (starting) stoichiometric BNT, loss of Bi_2_O_3_ during sample processing could lead to formation of oxygen vacancies. Starting compositions with Bi-deficiency (e.g., Bi_0.49_BT) have additional oxygen vacancies and therefore exhibit higher oxide ion conductivity, whereas starting compositions with excess Bi_2_O_3_ (e.g., Bi_0.51_NT) could suppress the oxygen and bismuth vacancy concentration and the oxide ion conductivity.

It is interesting to note that Na nonstoichiometry in the starting composition influences the electrical properties in a different way compared to Bi nonstoichiometry [102,103]. For example, compositions with Na deficiency are insulating, behaving similarly to compositions with Bi excess. Compositions with Na excess are conducting, behaving similarly to compositions with Bi deficiency. This appears confusing, as one expects the Na excess should help compensate A-site cation loss and suppress oxygen vacancies whereas Na deficiency should create oxygen vacancies and give higher oxide ion conductivity. SEM/TEM work on the presence of secondary phases clarified the origins of the apparent peculiar behavior. In the Na deficient composition (BNa_0.49_T), TEM results reveal the presence of TiO_2_ secondary phase [103]. By renormalizing the composition to Bi_0.51_Na_0.50_Ti_1.02_O_3.065_ and considering it as Bi_0.51_Na_0.50_Ti_1.02-x_O_3.055-2*x*_ + *x* TiO_2_ (*x* TiO_2_ represents the TiO_2_ secondary phase), it is apparent the BNa_0.49_T composition is effectively Bi-excess and therefore behaves similarly to Bi_0.51_NT. On the other hand, the Na-excess composition BNa_0.51_T can be considered as Bi-deficient due to Bi loss during sample processing and therefore behave similarly to Bi_0.49_NT.

It should be stressed the nonstoichiometry level in BNT is low. Secondary phases are observed in the nonstoichiometric starting compositions [102,103]. Compositional analysis using scanning electron microscopy—energy dispersive spectrometry (SEM-EDS) at a local (grain) scale and inductively coupled plasma—atomic emission spectroscopy (ICP-AES) on the overall composition [102] revealed no appreciable bulk compositional differences between these samples. It is found that even 0.5–1 at% Nb donor doping can fill the oxygen vacancies and suppress the oxide ion conductivity [67,102]. This suggests the nonstoichiometry in BNT is in the range of 0.0017–0.0033 for bismuth and 0.0025–0.0050 for oxygen. Clearly, the electrical properties of BNT are highly sensitive to low levels of nonstoichiometry. Similarly to that commonly observed in PZT, the generation/suppression of oxygen vacancies influence ferroelectric and piezoelectric properties including dielectric loss, coercive field, domain wall movement, fatigue, *T*_d_, d_33_, *etc.* Careful control of starting compositions and processing conditions is therefore crucible to obtain desirable and reproducible samples.

## 3. Polarization and Strain Curves

The phase diagrams of BNT and its various solid solutions (BNT-BT, BNT-BKT) manifest themselves in the polarization and strain curve in that way that well below the depolarization temperature *T*_d_ the polarization curve looks like a classical ferroelectric hysteresis curve. The same is true for the strain curve. It is a typical “butterfly” curve with a remanent strain. Approaching *T*_d_, the polarization curve gets pinched reminding on an antiferroelectric polarization curve. The remanent strain in the strain curves is reduced and the minima at coercive field become broader. Well above *T*_d_ the pinching of the polarization curve disappears and a very slim hysteresis curve develops, which is typical for a relaxor material. The strain curve completely loses its butterfly form and gets “sprout” shaped.

**Figure 6:**
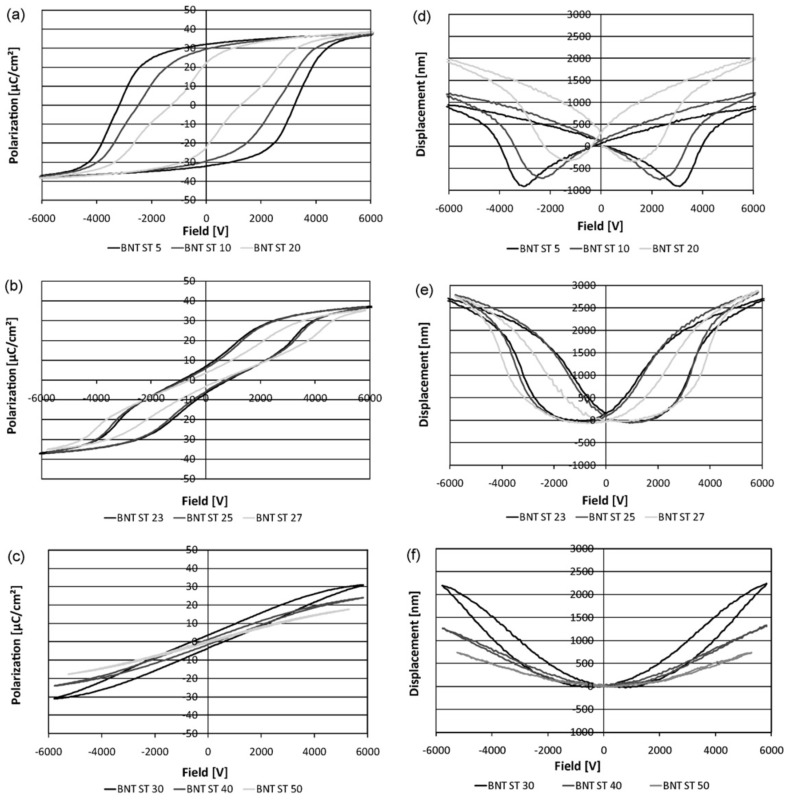
Polarization and displacement curves for the system (100−*x*)(Bi_0.5_Na_0.5_)TiO_3_–*x*SrTiO3 (x = 5, 10, 20 (**a,d**), x = 23, 25, 27 (**b,e**), x = 30, 40, 50 (**c,f**), reprinted from reference [109] with copyright from Elsevier 2010).

This shape change can be achieved either by increasing temperature or by changing chemical composition. Examples are nicely shown by Krauss, *et al.* [109] for the compositional change in the BNT-ST system (Figure 6) or by Wook Jo, *et al.* [110] for compositional and temperature change in the BNT-BT-KNN system. As can be seen from phase diagrams of BNT-BT [72] or BNT-BKT [111] *T*_d_ is minimized at regions addressed as morphotropic phase boundaries in solid solutions of rhombohedral BNT and a tetragonal compound (around 6 mol % BT or 17 mol % BKT). The substitution of A-site cations with Rare Earth cations such as Nd^3+^ also effectively decreases *T*_d_.

It is worth mentioning that the appearance of a pinched hysteresis loop in these materials is observed at temperatures up to 70 K lower than the denoted depolarization temperature in the phase diagram. Such phase diagrams are usually derived from temperature dependent capacitance and loss factor measurements under low signal AC conditions (e.g., 0.1–1 Vrms at 10 kHz [112]). *T*_d_ is assigned to a maximum in the loss factor, which shows pronounced frequency dispersion. This frequency dispersion is at least to some minor extent responsible for the abovementioned temperature difference because hysteresis curves are measured at much lower frequencies, e.g., 0.1 Hz. The major reason for the discrepancy is most probably the phase transition that is induced by large electric fields, which was shown clearly by Ma, *et al.* [91] for the BNT-BT solid solution. In this paper, this group enfolds a phase diagram of electric field *vs.* composition. In the range of 6–7 mol % BT it could be proven by combination of in-situ TEM structural analysis and large signal d_33_ measurements on bulk samples that the material undergoes an irreversible phase transition from a relaxor ferrielectric with P4bm symmetry into a mixed phase of R3c/P4mm symmetry, which is assigned ferroelectric, above 3 kV/mm. This mixed phase shows higher d_33_ values than the virgin P4bm phase. Thus, it can be stated, that the origin of the large field induced strain is a ferrielectric–ferroelectric phase transition generated by the external electric field. It was also shown that at 6 mol % BT this mixed phase can be destroyed at electric fields exceeding 5 kV/mm resulting in a single R3c phase with lower d_33_ values.

**Figure 7:**
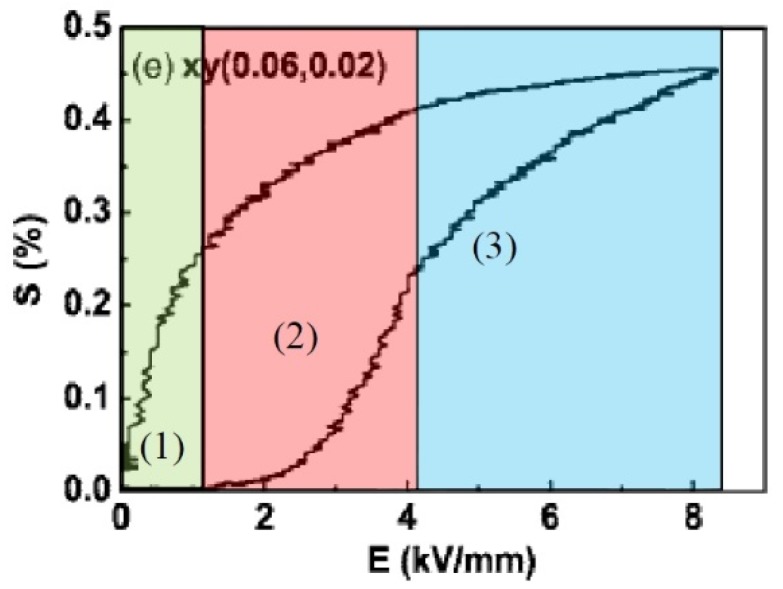
The positive part of the strain *versus* applied electric field loops for 92 BNT-6BT-2KNN. The regions indicated in the diagram are responsible for the peculiar electromechanical behavior and are related to a particular structure of the material: (1) low-strain region, weak ferroelectric; (2) electrostrictive region, relaxor ferrielectric; and (3) ferroelectric linear region (reprinted with permission from reference [18]. Copyright from AIP Publishing LLC 2008).

As an example of a typical strain curve (Figure 7) one is taken from the publication of Zhang, *et al.* [18] where the giant field induced strain was reported on the solid solution (1–*x*–*y*) [Bi_0.5_Na_0.5_TiO_3_] *x*[BaTiO_3_] y[K_0.5_Na_0.5_NbO_3_] for *x* = 0.06 and *y* = 0.02. A closer look reveals three distinct regions. Up to approximately 1 kV/mm, a low strain region (1) of a weak ferroelectric is observed with d_33_ values around 40 pm/V. Between 1 and 4 kV/mm, an electrostrictive region (2) with a second order curvature follows, which can be assigned to the relaxor ferrielectric mentioned above, and, Finally, at higher field, a ferroelectric part (3) exhibiting an almost linear (first-order) curve is observed, which fades out into saturation.

**Figure 8:**
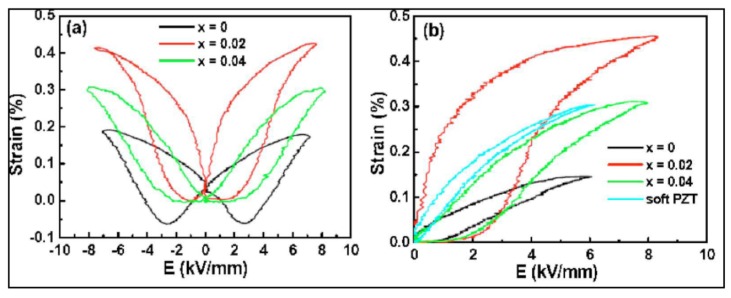
Bipolar (**a**) and unipolar (**b**) strain curves of (0.94−x)BNT-0.06BT-xKNN in comparison with a soft PZT (reprinted with permission from reference [113]. Copyright from AIP Publishing LLC 2007).

The studies of Zhang, *et al.* [113] proved that the strain of the system (1–*x*–*y*) [Bi_0.5_Na_0.5_TiO_3_] *x*[BaTiO_3_] *y*[K_0.5_Na_0.5_NbO_3_] for *x* = 0.06 and *y* = 0.02 exceeds the strain of a soft PZT by more than 50% (Figure 8). The lead-free system exhibits a maximum strain of 0.45% at 8 kV/mm *vs.* 0.3% for soft PZT, which saturated at 4 kV/mm. So promising this extraordinarily high strain is, the comparison also reveals the challenges, when replacing PZT with a BNT system in actuators. First, the maximum strain is achieved at much higher electric fields, which is an issue for lifetime prediction. Second, the area in the hysteresis loop is much larger, which means higher losses and a higher self-heating. The third issue is not obvious from Figure 8, but comes from the fact that this extraordinarily high strain is achieved for compositions operated at or near *T_d_*. This results in a high temperature dependence of the strain. In a later publication, Zhang, *et al.* [18] denoted for the given example at 180 °C less than half of the strain measured at ambient conditions. Finally there is the question about the work an actuator can render. This in fact is the product of strain and force. In the following, we discuss the features that need to be addressed for the design of a material used for actuator applications.

## 4. Texturing of Lead-Free Piezoelectric Ceramics

Texturing of ceramic microstructures is a process that promotes preferred orientation of grains (crystals) along specific crystallographic directions, the establishment of large anisometric grains and even the orientation of defects. This preferred orientation can either enhance or compromise the performance of ceramic components. Until the 1990s, most of the research onto textured ceramics was mainly focused on high temperature superconductors and ceramic components for structural applications. Lately, crystallographic texturing of ferroelectric ceramics has been regarded as an opportunity to exploit the anisotropic character of piezoelectric crystals and achieve significant enhancements in the piezoelectric response of Pb-free ceramics. For example, textured piezoceramics exhibit piezoelectric coefficients that can be two to three times higher than those shown by their randomly oriented counterparts and as high as 90% of the single-crystal values. Basically, the orientation of grains along preferred crystallographic directions promotes a more efficient alignment of polar vectors, leading to an increased poling efficiency and thereby an enhanced electromechanical response for a piezoceramic component. This is particularly beneficial for piezoelectrics where only two antiparallel states are allowed, because in microstructures encompassing randomly oriented grains, the percolation of the polarization is inhibited by grains which do not have possible domains states nearly aligned with the applied electric field. In principle those grains are not switchable and will prevent the switching of neighboring grains, which may actually have domains oriented more favorably with the applied field.

In the BNT–BT system, rhombohedral single-crystal can exhibit an electric-filed induced strain along the <100> as large as 0.25%, whereas tetragonal crystals show an even larger strain as high as 0.85%, but with large hysteresis along the same direction [114]. It is believed that the exceptionally high strain of the tetragonal crystals arises from domain switching. These giant strain values are suitable for actuator applications, such fuel injectors, therefore BNT-based ceramics with textured microstructures have attracted significant scientific and commercial interest in recent years.

The first proposal of texturing of piezoelectric dates back to the late 1960s, and it was suggested for SbSi. This proposal was then extended in the mid-1980s to bismuth-layered ferroelectrics and tungsten bronzes, however in the following years research into texturing of piezoelectric undergone little progress and it was not until the late 1990s that a promising method for texturing perovskite-structured ferroelectrics emerged. Indeed, in 1998, T. Tani [115] from the Toyota Central Research and Development Laboratories, proposed the fabrication of BNT by a reactive template grain growth (RTGG) method.

Other methods exist for the fabrication of textured piezoceramics, which can be categorized as follows: (1) oriented consolidation of anisometric particles (OCAP); (2) templated grain growth (TGG); and (3) heterotemplated grain growth (HTGG). For details on these techniques, the reader is referred to excellent reviews by Kimura [116] and Messing, *et al.* [117]. Nevertheless, it is worth to mention that all these methods tend to rely on sintering of green compacts containing pre-aligned anisometric particles. Currently, it is still difficult to obtain fully textured piezoelectric ceramics, because processing parameters have not yet been completely optimized, but texturing has been extensively employed for the development of Pb-free piezoelectric ceramics. Hereafter, we are reviewing chronologically the major advances achieved by texturing BNT-based piezoceramics.

As aforementioned the first attempt to induce texture in a BNT-based ceramic was conducted in 1998 by Tani [115], who employed the RTGG method to fabricate BNT ceramics. This process involves *in-situ* formation of a guest material (Bi_0.5_Na_0.5_TiO_3_, BNT) onto anisotropic oriented host particles (Bi_4_Ti_3_O_12_, BIT), a host-to-guest reaction, followed by grain growth to lead to a single-phase oriented microstructure. Basically, Tani [115] prepared large anisometric BIT particles (5 μm × 5 μm × 0.2 μm) by a molten salts route, and then mixed those with Bi_2_O_3_, Na_2_CO_3_ and TiO_2_ in ethanol and toluene. Green sheets containing aligned BIT were formed by tape casting and subsequently laminated. BNT was formed by *in-situ* reactions between BIT and the other starting materials. Tani [115] extended this process to the fabrication of textured Bi_0.5_Na_0.5_TiO_3_-15mol % Bi_0.5_K_0.5_TiO_3_ (BNT-BKT). BNT-BKT ceramics prepared by the RTGG method exhibited ~40% larger K_p_ values and ~60% larger *d*_31_ values when compared with non-textured ceramics of the same composition as shown in Table 1.

**Table 1:** Properties for BNT-BKT ceramics prepared by different methods.

Preparation Method	Conventional Sintering	RTGG Sintered	RTGG Hot-Pressed
Relative density (%)	99.2	97.0	98.6
Lotgering factor	0	0.92	0.90
ε_33_/ε_o_ (at 1 kHz)	593	595	644
tan δ (at 1 kHz)	0.025	0.014	0.013
Poisson’s ratio	0.250	0.180	0.176
*K*_t_	0.427	0.443	0.467
*K*_p_	0.295	0.402	0.431
*d*_31_ (pC/N)	36.7	57.4	63.1
*g*_31_ (10^−3^ Vm/N)	7.21	11.2	11.4

**Figure 9:**
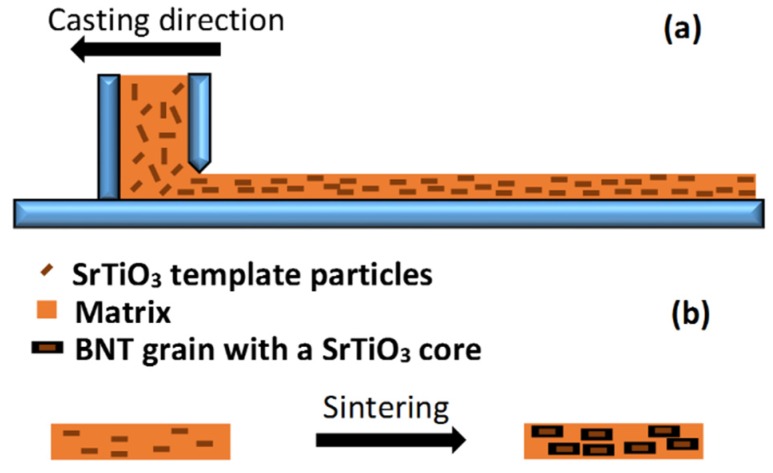
Schematics of template alignment by tape casting (**a**) and the texture fraction increases with heating (**b**) (based on reference [117]).

In 2000, Yilmaz, *et al.* [118] presented their preliminary results for [001] textured Bi_0.5_Na_0.5_TiO_3_-5.5 mol % BaTiO_3_ ceramics prepared by a TGG process. Basically, those researchers employed tape casting to orientate molten salt synthesized (001) SrTiO_3_ platelets in a BNT matrix, as schematically illustrated in Figure 9. During firing, Bi_0.5_Na_0.5_TiO_3_-5.5 mol % BaTiO_3_ nucleated and grew on the surface of the SrTiO_3_ templates, achieving a 94% textured microstructure. Later, these researchers also studied RTGG of Bi_0.5_Na_0.5_TiO_3_-5.5 mol % BaTiO_3_ using Bi_4_Ti_3_O_12_ template particles. In that case, a texture fraction of 80% was obtained, as measured by the Lotgering factor [119].

**Figure 10:**
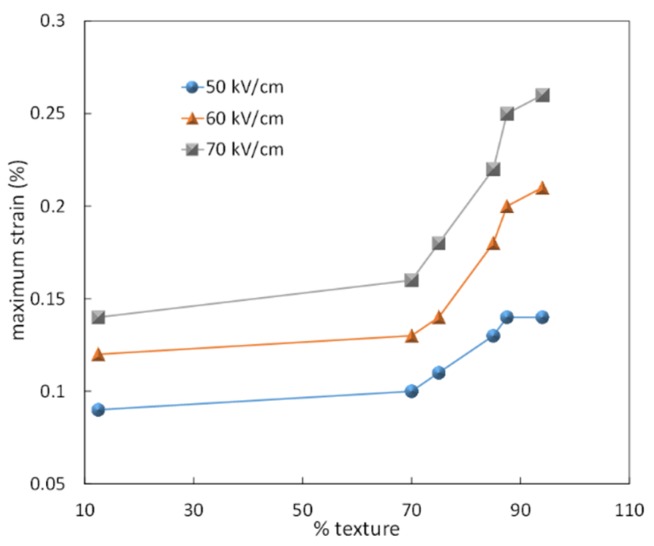
Unipolar Maximum strain of textured Bi_0.5_Na_0.5_TiO_3_-5.5 mol % BaTiO_3_ ceramics textured with 6 vol % SrTiO_3_ as function of the Lotgering factor.

The maximum unipolar strain of Bi_0.5_Na_0.5_TiO_3_-5.5 mol % BaTiO_3_ ceramics textured with 5.5 vol % SrTiO_3_ template particles as function of Lotgering factor, is illustrated in Figure 10. It is apparent that for texture fractions lower than 70%, the maximum strain is only moderately dependent on texture. Nevertheless, for texture fractions greater than 70% the maximum strain appears to increase significantly. For example, a sample with 94% texture shows 0.26% strain when measured at 70 kV/cm [119].

In 2002, the RTGG process for Bi_0.5_(Na_1–*x*_K*_x_*)_0.5_TiO_3_ was studied in detail by Fukuchi, *et al.* [120], who proposed the following mechanism when starting with plate-like particles of BIT prepared by a molten salts route. Calcination promotes *in-situ* reactions as follows:

Bi_4_Ti_3_O_12_ + 2(1–*x*)Na_2_CO_3_ + 2*x*K_2_CO_3_ + 5TiO_2_ → 8BNKT + 2CO_2_ (2)

and

Bi_2_O_3_ + (1–*x*)Na_2_CO_3_ + *x*K_2_CO_3_ + 4TiO_2_ → 4BNKT + CO_2_ (3)

**Figure 11:**
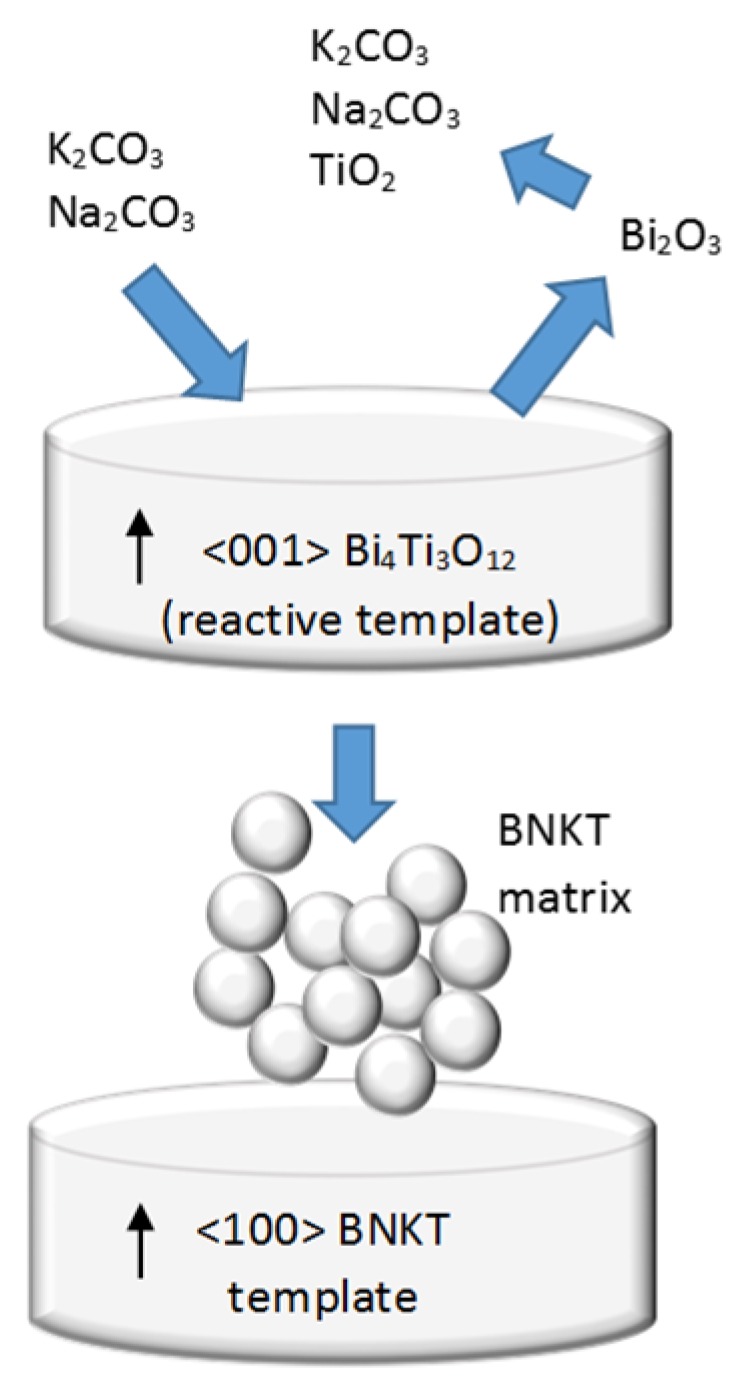
Schematic representation for the formation of BNKT templates from BIT particles and complementary reactants.

Equation (2) forms plate-like BNKT particles (templates) with the crystallographic <100> direction perpendicular to the plate face and Equation (3) forms equiaxed BNKT particles (matrix) with random orientation. The oriented template particles grow at the expense of the matrix particles during sintering. In an alternative explanation, plate-like BNKT particles are formed due to Na_2_O and K_2_O diffusion into the BIT lattice, whereas small equiaxed BNKT particles (matrix) are formed by the reaction of K_2_CO_3_, Na_2_CO_3_ and TiO_2_ with Bi_2_O_3_ that diffuses out of BIT, as schematically illustrated in Figure 11.

In 2003, West and Payne [121] investigated both template and formulation effects on the microstructural development of BNT-based ceramics made by the RTGG method. Basically, they aimed to investigate the impact of both crystallography and chemistry of the template phase on the RTGG process. For this purpose they prepared Sr_3_Ti_2_O_7_ (Ruddlesden–Popper structured) and BaBi_2_Nb_2_O_9_ (Aurivillius structured) plate-like “templates” using a molten salts route, which were subsequently mixed with fine particles of BNT. It was found that perovskite formation was more sluggish in the mixtures templated with Sr_3_Ti_2_O_7_, and the final sintered microstructure featured larger, porous grains in an equiaxed, micrometer-sized matrix. They also postulated that successful RTGG processing of the BNT-based using Aurivillius templates could be achieved for matrix whose formulation contained excess alkali.

According to Kimura, *et al.* [122], there are two ways to develop crystallographic texture in BNT. One involves the addition of an excessive amount of Bi_2_O_3_ to promote preferential growth of oriented grains. Basically, in stoichiometric BNT, the growth rate of matrix grains is high and the conditions for the preferential growth of the oriented grains are disrupted. Nevertheless, excess Bi_2_O_3_ promotes preferential growth of oriented grains leading to the development of highly textured BNT. These textured BNT ceramics exhibit 70% higher piezoelectric *d*_31_ and electromechanical *k*_p_ coefficients than non-textured BNT. A more effective way to achieve highly textured BNT microstructures is to increase the amount of plate-like BIT in the initial formulation. Kimura, *et al.* [122,123] prepared BNT and Bi_0.5_Na_0.5_TiO_3_-6 mol %BaTiO_3_ bulk ceramics with extensive <100> texture by the RTGG method, using plate-like BIT particles as templates for BNT. Because only BIT contributes to the formation of oriented grains, in order to increase volume of oriented grains the initial formulation needs to be modified, e.g., according to the following reaction

BIT + 2Na_2_CO_3_ + 5TiO_2_ = 8BNT + 2CO_2_ (4)

It is possible to supply 37.5% Ti in the final product from plate-like BIT, and obtain highly textured BNT. Kimura, *et al.* [123] extended this formulation to prepare successfully prepare textured Bi_0.5_Na_0.5_TiO_3_-6 mol % BaTiO_3_ ceramics. Interestingly the selection of BaTiO_3_ source (BaTiO_3_ or BaCO_3_ + TiO_2_) plays a remarkable role; BaTiO_3_ has little adverse effect on the texture development but BaCO_3_ + TiO_2_ reduces the volume of oriented grains. Hence, texturing in BNT-BT ceramics is easily achieved if BaTiO_3_ is used as the source.

In 2006, Fuse and Kimura [124] studied the effect of particle sizes of starting materials on microstructure development in textured Bi_0.5_(Na_0.5_K_0.5_)_0.5_TiO_3_. During sintering texture develops by the growth of template grains at the expense of matrix grains. This grain growth rate is determined by the template and matrix grain sizes. Hence, textured Bi_0.5_(Na_0.5_K_0.5_)_0.5_TiO_3_ ceramics with a large degree of orientation and homogeneous microstructures are obtainable by the combination of small or medium BIT and small TiO_2_. Nevertheless, if the use of large BIT is desirable, textured ceramics without matrix grains are obtained by increasing the amount of BIT in the starting formulation. This is because the size of grains in the final microstructure is determined by the BIT particle size.

Researchers also started looking for alternative templates to BIT. For example, in 2006 Zeng, *et al.* [125] synthesized large plate-like BNT templates from the bismuth layer-structured ferroelectric Na_0.5_Bi_4.5_Ti_4_O_15_ (NBIT) particles. Owing to the similarity in structure between BNT and NBIT, it is possible to transform the layer-structured NBIT to a perovskite BNT by a topochemical method. Hence, large highly anisometric NBIT particles are first synthesized by the molten-salt process. After the topochemical reaction with the complementary reactants (Na_2_CO_3_ and TiO_2_) in NaCl flux, the layer-structured NBIT particles are completely transformed into BNT, with retention of the plate-like morphology. These authors suggested these BNT templates to be effective at inducing grain orientation in the BNKT-BT ceramics in comparison with BIT templates. For a BNKT-BT ceramic textured with 20 wt % of BNT templates, they measured a Lotgering factor of 0.89. The textured BNKT-BT ceramics templated by BNT showed *d*_33_~215 pC/N compared with 167 pC/N for randomly oriented ceramics. Moreover, textured BNKT-BT ceramics templated by BIT show lower *d*_33_~120 pC/N, mainly due to their lower density. Zhao, *et al.* [126,127] further corroborated the suitability of high-aspect-ratio BNT templates to obtain textured ceramics (especially BNT-based ceramics) by RTGG. For BNT-6 mol % BaTiO_3_ ceramics textured with 5 vol % BNT templates, they obtained a Lotgering factor of 0.87 and a *d*_33_ of 299 pC/N.

In 2008, Gao, *et al.* [128] employed one-dimensional needle-like KSr_2_Nb_5_O_15_ (KSN) and two-dimensional plate-like Bi_2.5_Na_3.5_Nb_5_O_18_ particles, as templates to fabricate textured Bi_0.5_Na_0.5_TiO_3_-BaTiO_3_ ceramics by the RTGG method. They found that that KSN was unsuitable to fabricate textured ceramics, because the KSN particles were aligned randomly along the tape casting direction. In contrast, textured ceramics with orientation factor more than 60% could be successfully obtained using BNN particles as templates. Moreover, texture fraction increased with increasing BNN content, as expected.

**Figure 12:**
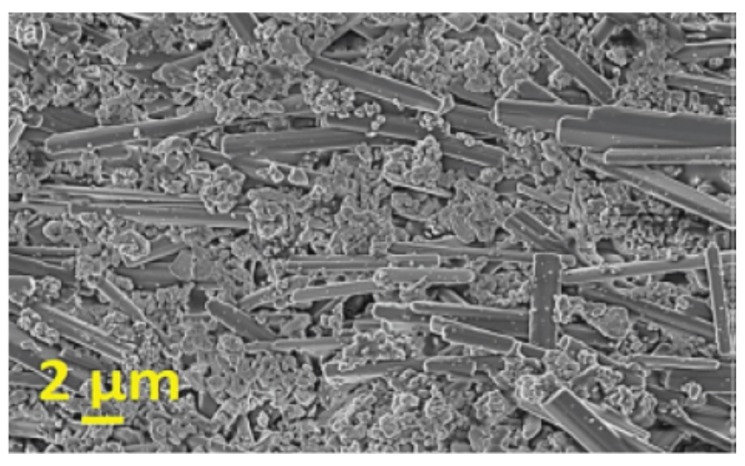
SEM image a single tape containing well-aligned Na_2_Ti_6_O_13_ template whiskers embedded in the base matrix powder [129].

In 2011, Maurya, *et al.* [129] use Na_2_Ti_6_O_13_ template whiskers prepared by molten salts for the preparation of textured BNT-7 mol % BaTiO_3_ ceramics. Figure 12 shows a single tape of matrix containing well-aligned Na_2_Ti_6_O_13_ template whiskers. The highest *d*_33_~216 pC/N was achieved for ceramics fired at 1200 °C.

In 2013, these researchers [130] synthesized again BNT–7 mol % BaTiO_3_ but employing BNT seeds. They obtained ceramics with 92% textured microstructures, which exhibited a 200% improvement *d*_33_ ~322 pC/N, as compared to 160 pC/N for its randomly oriented counterparts. Later, Maurya, *et al.* [131] also fabricated grain-oriented lead-free ceramics from the Bi_0.5_K_0.5_TiO_3_-BaTiO_3_-xBi_0.5_Na_0.5_TiO_3_ (BKT-BT-BNT) system with high degree of texturing along the [001]_c_ crystallographic orientation. In this case, they employed BaTiO_3_ seeds as the template particles. The textured specimens had depoling temperatures of more than 165 °C, revealing a higher stability temperature for lead-free piezoelectric systems. Moreover, textured ceramics exhibited a ~70% increase in *d*_33_ (~190 pC/N), and more than 200% increase in the *E*-field induced strain (0.44% as compared to 0.23% in non-textured ceramics) with a ultra-low degree of hysteresis.

In 2014, Hu, *et al.* [132] used a layered titanate H_1.07_Ti_1.73_O_4_·nH_2_O (HTO) with a plate-like particle morphology as an alternative template to BIT or BNT for the fabrication of <100> oriented BNT ceramics by RTGG. Nevertheless the improvement of the piezoelectric performance appeared to be modest.

Very recently, Hussain, *et al.* [133] prepared 0.94Bi_0.5_Na_0.5_TiO_3_-0.06BaZrO_3_ (BNT-BZ) ceramics by a RTGG method using 15 wt % BNT as template particles. The as-synthesized BNT-BZ textured ceramics were sintered at 1150 °C for different firing times (2–15 h). These researchers observed that all textured BNT-BZ ceramics have (h00) preferred orientation, which increased with increasing sintering times. The depolarization temperature was unaltered by the sintering time. However, the field induced strain response increased by 20% when the sintering time was increased from 2 h to 15 h.

**Figure 13:**
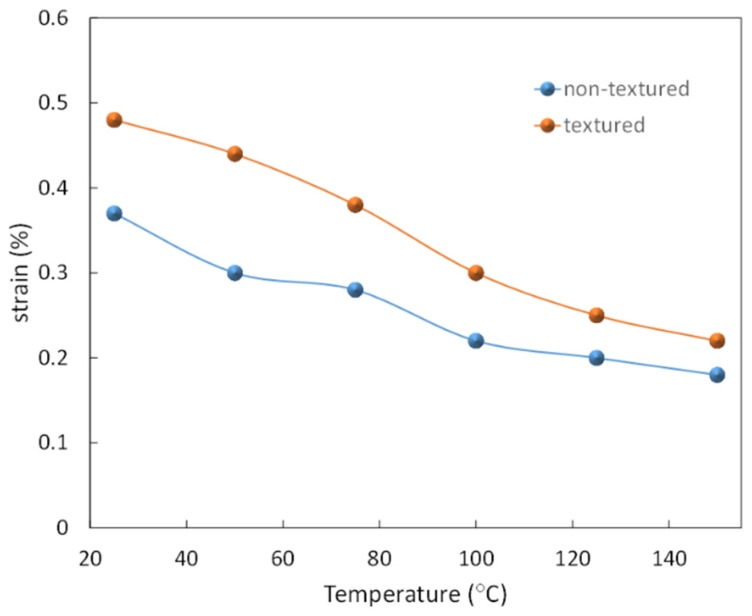
Temperature dependence of the strain for 0.91Bi_0.5_Na_0.5_TiO_3_–0.06BaTiO_3_–0.03AgNbO_3_ textured *vs.* non-textured ceramics (After Zhang, *et al.* [134]).

In addition, in 2015, Zhang, *et al.* [134] prepared by <100>-oriented 0.91Bi_0.5_Na_0.5_TiO_3_–0.06BaTiO_3_–0.03AgNbO_3_ ceramics with a Lotgering factor of 0.71 using plate-like BNT templates. A high unipolar strain of 0.38% and a large signal piezoelectric coefficient of 766 pm/V at 5 kV/mm was measured for these textured ceramics, which are 78% higher than the randomly oriented samples. It was found that textured ceramics exhibited significantly reduced frequency dependence in the unipolar strain behavior at room temperature, resulting from the decreased electric field required for the relaxor-ferroelectric phase transition. Both textured and randomly oriented ceramics have zero negative strains, suggesting the giant strain originated from a reversible electric field-induced phase transition, as expected for an incipient piezoelectric. With increasing temperature, increasing strain hysteresis almost vanishes and the induced ferroelectric state becomes negligible. Above 100 °C, there is no electric field-induced phase transition with the applied electric field and therefore the electrostrictive response is the dominant contribution to the strain, which becomes similar to both ceramics. Temperature dependence of the strain for 0.91Bi_0.5_Na_0.5_TiO_3_–0.06BaTiO_3_–0.03AgNbO_3_ textured *versus* non-textured ceramics is illustrated in Figure 13.

Key features of the aforementioned developments in textured microstructures for BNT-based ceramics are summarized in Table 2.

**Table 2:** Table illustrating the impact of texturing on the piezoelectric coefficients of BNT-based ceramics.

BNT-Based System	Template Type	Lotgering Factor (%)	Small Signal Piezoelectric Coefficient (pC/N)	Reference
BNT-15BKT (hot-pressed)	Bi_4_Ti_3_O_12_	~90	*d*_31_ = 63.1	Tani [115]
BNT-5.5BT	SrTiO_3_	~94	*d*_33_ = 200	Yilmaz, *et al.* [119]
BNT-15BKT	Bi_4_Ti_3_O_12_	~90	*d*_31_ = 50	Fukuchi, *et al.* [120]
BNT-6BT	Bi_4_Ti_3_O_12_	~90	*d*_31_ = −31.4	Kimura, *et al.* [122]
BNKT-BT	Bi_0.5_Na_0.5_TiO_3_	~89	*d*_33_ = 215	Zheng, *et al.* [125]
BNT-6BT	Bi_0.5_Na_0.5_TiO_3_	~87	*d*_33_ = 299	Zhao, *et al.* [126]
BNT-7BT	Na_2_Ti_6_O_13_	~7	*d*_33_ = 216	Maurya, *et al.* [129]
BNT-7BT	Bi_0.5_Na_0.5_TiO_3_	~92	*d*_33_ = 322	Maurya, *et al.* [130]
BNT-BT-BKT	BaTiO_3_	~93	*d*_33_ = 190	Maurya, *et al.* [131]
BNT-6BZ	Bi_0.5_Na_0.5_TiO_3_	~83	*d*_33_ = 22	Hussain, *et al.* [133]
BNT-6BT-3AN	Bi_0.5_Na_0.5_TiO_3_	~71	*d*_33_ = 766 at 5 kV	Zhang, *et al.* [134]

## 5. Features of Actuator Materials

### 5.1. Strain

The strain over electric field of a soft PZT typically shows a butterfly curve. Usually an actuator is operated in unipolar mode. In this operation mode only the difference between maximum strain and remanent strain can be used for actuation. In rare cases an electric field of opposite polarity is applied well below the coercive field in order to increase the usable strain. A bipolar operation is prohibited because of the extremely high stresses that occur during reversal of polarity and a tremendous self-heating because of the intrinsic losses. The sprout shaped strain curve of a BNT based material near the depolarization temperature has a minimal remanent strain. Therefore the usable strain for actuation is much higher than in the case of a classical ferroelectric. In addition, a bipolar operation would be conceivable. However, as mentioned before, strain alone is not enough for the operation of an actuator. It is more the product of strain and force that expresses the energy or work that an actuator can deliver. Having in mind an actuator stack for opening the diesel injection valve in a combustion engine [135,136], this device makes a stroke of just about 40 μm but against a fuel pressure of up to 2000 bar. Further, it has turned out advantageous to operate such a stack under pre-stress conditions, *i.e.*, the device is mounted in a spring-like housing that exerts a load of about 30 MPa to the stack [137]. This can optimize the usable strain and prevents disintegration by cracking.

### 5.2. Blocking Force

Usually two figures are given for a piezoelectric actuator, the free strain and the blocking force. The free strain is the strain on an actuator without any external load (technically users sometimes measure and refer to the free stroke, which should be normalized to the free strain to eliminate geometry factors). Usually it depends on the applied voltage in a linear manner up to the saturation. The blocking force is the maximum force an actuator can generate in clamped state (zero displacement) [138]. Since the blocking force also depends on the geometry of the actuator it is reasonable to refer to the blocking stress. Although both values are often cited to define the performance and work output of an actuator, it is obvious that in both cases the energy conversion is zero. To characterize the performance under mechanical load a rather sophisticated measurement setup has to be installed. In many cases, a good approximation of a strain/stress-diagram is obtained when the free strain and the blocking stress are connected by a straight line.

A careful measurement of blocking stress and energy conversion on BNT-based materials was performed by Dittmer, *et al.* [139]. The group selected BNT-6BT representing a ferroelectric at the morphotropic phase boundary similar to PZT, and BNT-6BT-2KNN, which can be considered a relaxor type material. The investigation included the dependence of free strain and blocking stress on electric field as well as on temperature. At room temperature, a linear increase of these two parameters with the applied field was observed for BNT-6BT. At 3 kV/mm a free strain of 0.064% and a blocking stress of 39 MPa was measured yielding a maximum specific work of 3.5 kJ/m^3^. This is much less than that of a soft PZT where a free strain of 0.19% and a blocking stress of 65 MPa is obtained under the same electric field yielding a six times higher specific energy. The performance of BNT-6BT is rapidly increased by increasing temperature peaking at 125 °C. Here free strain and blocking stress reach 0.26% and more than 100 MPa, respectively. Consequently, the specific energy yields 39 kJ/m^3^, which significantly exceeds that of PZT, whose properties are much more temperature stable and increase about 10%–20% up to 150 °C.

**Figure 14:**
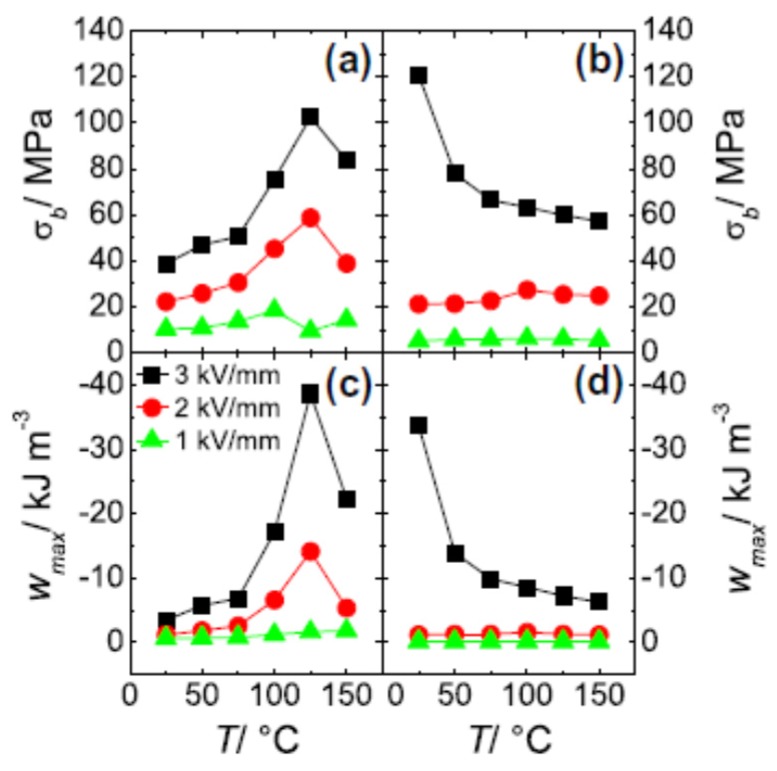
Blocking stress and maximum specific work for BNT-6BT (**a**,**c**); and for BNT-6BT-2KNN (**b**,**d**) as a function of temperature (reprinted from Reference [139] with copyright from Elsevier 2012).

On the other hand, BNT-6BT-2KNN shows a strongly nonlinear increase of free strain and blocking stress with increasing electric field, which is due to the field induced phase transition that occurs in this material already at room temperature. The values at 3 kV/mm (0.21% free strain and 124 MPa blocking stress) exceed that of BNT-6BT and PZT by far. The maximum specific work of about 35 kJ/m^3^ is three times higher than that of PZT. However, with increasing temperature these values decrease rapidly. Figure 14 illustrates the temperature and field dependence of free strain and blocking stress of these two materials. It makes clear that the temperature range where their properties exceed that of PZT is rather narrow. Therefore, efforts were undertaken to broaden this range. This topic will be discussed in the following section.

Investigation of strain curves under variable stress [140] was carried out on multilayer stacks consisting of 50 single layers made of BNT-BKT-BLT ceramics doped with 2 mol % Nd, a material already optimized for increased temperature stability of electromechanical properties [141]. For the electromechanical characterization, a constant stress varying between 0.1 and 80 MPa was applied on the stack and subsequently five unipolar voltage ramps corresponding to a maximum electric field of 8 kV/mm were applied. During the voltage cycling the stress was kept constant and the strain was measured.

**Figure 15:**
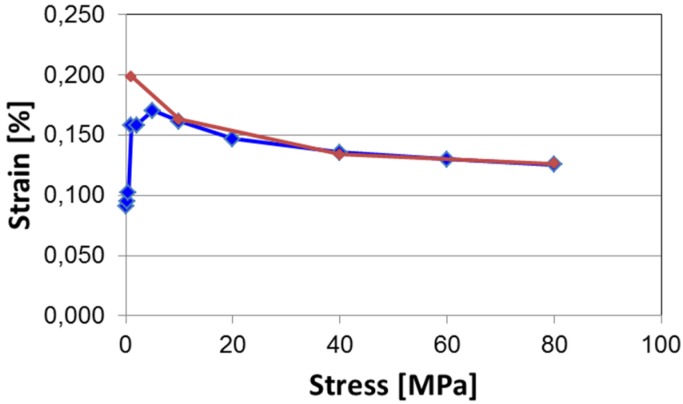
Effective strain of a stack actuator made of Nd-doped BNT-BKT-BLT at various stress levels at 8 kV/mm (blue symbols: increasing stress, red symbols: decreasing stress).

Figure 15 depicts the effective strain of this stack depending on the applied stress. In the virgin sample an applied stress of only 0.1 MPa reduces the strain to less than 0.1%, which is roughly half of the free strain measured with a laser interferometer. Increasing stress leads to an increase of effective strain peaking at 5 MPa with 0.17% (blue symbols in Figure 15). A further increase of the stress up to 80 MPa slightly decreases the effective strain. When decreasing stress, the measured strain values follow the curve obtained under increasing stress conditions down to 5 MPa. Below this stress level the strain does not follow the initial curve but further increases and reaches the level of free strain at 1 MPa.

This work revealed that such a material operating in the relaxor regime is capable of excerting a strain of 0.125% under a stress of 80 MPa, which is equivalent to a load of 3300 N. This is comparable to commercial PZT based actuators used in fuel injection systems. The development of strain under increasing and decreasing stress gives evidence for a mechanically induced and irreversible transition.

### 5.3. Temperature Dependence of Strain

**Figure 16:**
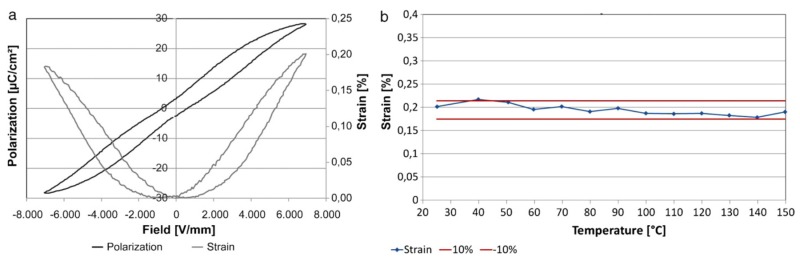
Polarization and displacement of a multilayer stack described by Krauss, *et al.* [141] at room temperature (**a**). Strain of the device at 7 kV/mm *vs.* temperature (**b**). Reprinted from [141] with copyright from Elsevier 2011.

As mentioned above, BNT based ceramics show distinct maxima in strain at the so-called depolarization temperature Td as shown in the examples in the section “Blocking Force”. Krauss, *et al.* [141] demonstrated on multilayer stacks with a BNT based material how to minimize the temperature dependence of strain. The approach was based on the concept to operate the actuator between depolarization temperature *T*_d_ and the temperature of maximum relative permittivity *T*_m_. This can be achieved by substitution of Bi and/or Na by smaller ions [16] or Ti by larger ions [142]. Obviously, such a substitution causes an increase of the A-site volume, which facilitates the phase transitions mentioned before [91]. Further solid solutions with tetragonal compounds like BaTiO3 [72,112] or BKT [73,143] lead to a minimum in T_d_ at the morphotropic phase boundary. Following these considerations the composition [Bi_0.50_Na_0.335_K_0.125_Li_0.04_]TiO_3_ doped with 2 mol % Neodymium was selected as ceramic material for the actuator stack. It is based on a BNT-BKT solid solution. The addition of Li and Nd increases the difference between *T*_d_ and *T*_m_. Figure 16a shows a narrow relaxor like polarization curve and a “sprout” shaped displacement curve with a maximum strain of 0.19% at 7 kV/mm. This strain exhibits a variation of only 10% between 25 and 150 °C (Figure 16b).

### 5.4. Degradation

After extensive studies over the past two decades, optimized BNT-based compositions have been developed to compete well with PZT in certain areas. For commercial applications, these compositions must simultaneously have reliable performance for the required lifespan. Ferroelectric BNT-based materials exhibit higher coercive field than PZT. Performance degradation during electric loading is a concern for practical applications [144,145,146]. Indeed, for the composition 0.94Bi_1/2_Na_1/2_TiO_3_–0.06BaTiO_3_ (94BNT–6BT), which resembles a morphotropic phase boundary composition, a significant degradation in remanent polarization, strain, piezoelectric constant *d*_33_ and relative permittivity ε_33_ is observed even within the first 10^4^ cycles [147]. Micro-cracks are observed near the electrode region after 10^4^ cycles of bipolar cycling, which are considered to contribute significantly to the degradation of the ferroelectric properties. Degradation and cracking are reported to be suppressed by addition of 1 at % CuO [148], which is attributed to either reduced defect charges (as demonstrated by lower conductivity) or reduced amount of rhombohedral phase and increased tetragonal phase fraction. It is considered that the rhombohedral phase is the main contributor to the macroscopic properties due to both 180° and non-180° domain switching. In the tetragonal phase, the 90° domain switching is suppressed due to the constraints of neighboring grains. Higher tetragonal phase fraction therefore leads to better fatigue resistance [148].

In one report, addition of KNN to BNT-BT (0.91(Bi_1/2_Na_1/2_)TiO_3_–0.06BaTiO_3_–0.03(K_0.5_Na_0.5_)NbO_3_) is shown to improve the fatigue resistance [149]. However, a subsequent report [150] shows this composition suffers rapid degradation. Defective electrodes or defects within the ceramic rather than cracks resulting from high strain output are considered as the origins of degradation. It is interesting to note that the composition 0.92Bi_1/2_Na_1/2_TiO_3_–0.08BaTiO_3_ (92BNT–8BT) has higher tetragonal phase fraction and exhibits good fatigue resistance [150]. The decrease of strain is only 10% after 10^8^ cycles for the 92BNT–8BT ceramics. Bi(Zn_0.5_Ti_0.5_)O_3_ (BZN) is reported to enhance fatigue resistance in the BNT-BT system [151] and BNT-BKT system [152].

### 5.5. Interaction with Electrode

High performance actuators operating at convenient voltage levels are usually manufactured by multilayer technology [135], which enables reducing ceramic layer thickness well below 100 μm. This technology includes co-firing of metal-ceramic-laminates, which strongly needs a good chemical and mechanical compatibility of these two constituents. Highly inert metals like Platinum might be appropriate for demonstrator devices but are commercially hardly enforceable.

Most actuator devices based on PZT use silver-palladium alloys as inner electrodes. The silver-palladium ratio determines the melting point that has to be adjusted to the sintering temperature. Metal-ceramic interaction comprises the diffusion of the electrode material into the ceramic and the formation of secondary phases at the metal ceramic interface leading to changes in the electrical properties of the ceramics or to delamination and loss of contact. In silver-palladium alloys the oxidation of palladium is observed between 300 and 835 °C constituting a secondary phase that can react with the ceramic. Further, the Ag/Pd ratio of the metal alloy is shifted to higher Ag-content causing a higher mobility and volatility of Ag. Another issue is the volume expansion, which can reach 15% [135,153]. The amount of PdO formed can be minimized by increasing the heating rate in the relevant temperature range.

PdO together with Bi_2_O_3_ forms the compound Bi_2_PdO_4_ in the temperature range between 350 °C and 835 °C [154,155]. After decomposition at 835 °C, even the formation of the intermetallic compound Pd*_x_*Bi*_y_* was observed. Thus, the use of silver-palladium alloy together with Bismuth-based ceramics was generally excluded and the first multilayer sample described by Nagata, *et al.* [156] contained platinum electrodes. In a study by Schütz, *et al.* [157] it was shown that the bismuth-palladium reaction does not take place if the presence of free Bi_2_O_3_ in the ceramic is avoided. This encouraged the use of silver palladium alloys for BNT-based multilayer devices.

To reduce the mechanical stress in multilayer devices during co-firing due to different onset of shrinkage and different thermal expansion coefficient the addition of ceramic powder to the electrode paste was used already for multilayer ceramic capacitors based on Barium Titanate. There usually sol-gel derived Barium Titanate powder with grain size around 100 nm is part of the nickel paste for inner electrodes. Such a concept for composite electrodes was suggested for lead free multilayer actuators based on BNT-BKT solid solution by Nguyen, *et al.* [158]. They applied a composite electrode consisting of Silver-Palladium alloy and powder of Potassium-Sodium-Niobate doped with Lithium and Tantalum. They observed the reduction of bending and cracking of the multilayer devices due to thermal mismatch and a three times higher normalized strain. Recently, Ahn, *et al.* [159] reported a significant reduction of sintering temperature for a BNT-BKT solid solution by addition of Copper Oxide from 1150 °C to 950 °C. This reduction in sintering temperature decreases the thermal stress for multilayer components and therefore has a potential to minimize mechanical and chemical interaction. Moreover, the Palladium content of the inner electrode can be reduced, which significantly lowers materials cost.

## 6. Summary

Bismuth-Sodium-Titanate based solid solutions are interesting lead-free materials for actuator devices because of their extraordinarily high strain due to a phase transition induced by electric field. After the discovery of this effect, researchers worked for nearly one decade on revealing the structural features of these compounds and the peculiarities of the electromechanical behavior. It could be demonstrated that the observed strain can be used for effective work by determining blocking force, stress–strain-curves and the strain development under varying mechanical load. Texturing techniques have been developed that enable increasing the electromechanical coupling factor. For the application as actuators it was important to design and characterize multilayer devices containing inner electrodes. It could be shown that Silver-Palladium alloys, standard electrodes in multilayer ceramic components, can be used with BNT-based ceramics. All these findings are a good basis for the development of commercial devices. Still, to our best knowledge, there is only one company offering a BNT-based ceramic material and bulk actuator components made thereof (PI Ceramic, Lederhose, Germany). One of the reasons might be the fact that the investigation of long-term stability and degradation of these materials is still at the beginning and needs to be deepened in order to provide sufficient understanding for the development of reliable products, especially because such actuator devices most probably have to be operated at higher electric fields compared to devices based on PZT. This issue together with some cost factors (more expensive and challenging materials synthesis because of the water-soluble alkali compounds, noble metal electrodes) makes many industrial partners hesitate in the commercial use of these materials and the replacement of the well established PZT.
